# Validation of clinical exome sequencing in the diagnostic procedure of patients with intellectual disability in clinical practice

**DOI:** 10.1186/s13023-023-02809-z

**Published:** 2023-07-21

**Authors:** María Juliana Ballesta-Martínez, Virginia Pérez-Fernández, Vanesa López-González, María José Sánchez-Soler, Ana Teresa Serrano-Antón, Lidia Isolina Rodríguez-Peña, Maria Barreda-Sánchez, Lluís Armengol-Dulcet, Encarna Guillén-Navarro

**Affiliations:** 1 Sección de Genética Médica, Servicio de Pediatría, Hospital Clínico Universitario Virgen de la Arrixaca, Murcia, Spain; 2 Instituto Murciano de Investigación Biomédica (IMIB), Murcia, Spain; 3 Centro de Investigación Biomédica en Red-Enfermedades Raras (CIBERER-Instituto de Salud Carlos III), Madrid, Spain; 4 Quantitative Genomic Medicine Laboratories (qGenomics), Esplugues del Llobregat, Catalonia Spain; 5 Departamento de Ciencias Sociosanitarias-Área de Bioestadística, Facultad de Medicina, Universidad de Murcia, Murcia, Spain

**Keywords:** Intellectual disability, Global developmental delay, Exome sequencing, NGS, Clinical exome sequencing, Diagnostic yield, Efficiency

## Abstract

Intellectual disability (ID) has a prevalence of 1–3% and aproximately 30–50% of ID cases have a genetic cause. Development of next-generation sequencing has shown a high diagnostic potential. The aim of this work was to evaluate the diagnostic yield of clinical exome sequencing in 188 ID patients and the economic impact of its introduction in clinical practice. An analysis of diagnostic yield according to the different clinical variables was performed in order to establish an efficient diagnostic protocol for ID patients. Diagnostic yield of clinical exome sequencing was significant (34%) supporting its utility in diagnosis of ID patients. Wide genetic heterogeneity and predominance of autosomal dominant de novo variants in ID patients were observed. Time to diagnosis was shortened and diagnostic study costs decreased by 62% after implementation of clinical exome sequencing. No association was found between any of the variables analyzed and a higher diagnostic yield; added to the fact that many of the diagnoses weren’t clinically detectable, the reduction of time to diagnosis and the economic savings with respect to classical diagnostic studies, strengthen the clinical and economical convenience of early implementation of clinical exome sequencing in the diagnostic workup of ID patients in clinical practice.

**Supplementary Information:**

The online version contains supplementary material available at 10.1186/s13023-023-02809-z.

## Introduction

Intellectual disability (ID) has a prevalence of 1–3% and affects the individual, its family and the community, therefore being a public health concern [1–6]. Approximately 30–50% of ID cases are due to a genetic cause, and 25–30% of these are due to genetic variants in single genes [2, 7, 8]. Achieving a molecular diagnosis in ID has an economic impact not only on the patient’s healthcare system, but also on their families, secondary to genetic counselling with prevention of new cases. In the same way a diagnosis also helps at educational and social levels [2, 4].

In the recent years there has been a massive description of new ID-related genes, new phenotypes, as well as identification of mild or atypical phenotypes of known clinical entities. These facts highlight the challege of clinical detection and the complexity towards a targeted molecular approach of ID cases. Development of next-generation sequencing (NGS) tecniques, which allow genome sequencing (GS), or exome sequencing (ES) or other diagnostic approaches such as clinical exome sequencing (sequencing of around 5.000 morbid genes described in OMIM associated to clinical phenotypes), have shown a high diagnostic capacity [8–14].

The diagnostic impact of NGS techniques in genetic diseases has been previously described in the literature, with yields around 25–46% in the case of intellectual disability patients [6, 10, 11, 14–20]. Other articles have remarked the effectiveness of the incorporation of NGS in the diagnostic workup of ID patients with considerable savings [4, 21–23]. Despite of this superior diagnostic capacity, after the application of these technologies, still around 50% of ID patients remain without a molecular diagnosis [2, 4, 11, 15, 16].

In this study we report on the diagnostic yield of clinical exome sequencing in a cohort of 188 ID patients. We describe the costs of the diagnostic procedure and analyze the impact of the incorporation of clinical exome sequencing as a first-tier tool for the analysis of ID patients.

## Materials and methods

This is a retrospective observational study of 188 ID patients in which clinical exome sequencing was performed as main part of the genetic diagnostic workup. In order to obtain a representative sample of ID population in this study, according to ID prevalence of 1–3% 3], and on the demographic data of the average population in Murcia in the years of study (2015–2018) of 1.470.000 people, we calculated a sample size of 180 patients with a precision of 2.5%.

All patients had undergone a complete clinical evaluation in the Clinical Genetics Section of the Hospital Clínico Universitario Virgen de la Arrixaca, Murcia, Spain, in order to exclude possible non-genetic causes for ID. Inclusion criteria also included a normal chromosomal microarray analysis (CMA) and fragile X molecular analysis. ID patients with a straightforward clinical diagnosis, with a genetic diagnose obtained after direct sequence analysis of the candidate gene, were not included.

Period of inclusion was from 2015 to 2018. In this period patient inclusion was also classified according to a *moment of evaluation* criteria. Patients who were evaluated for the first time during this period were classified as *new patient* group. Another group of patients were those who had been evaluated in previous years without achieving a diagnosis (before incorporation of NGS in the diagnostic workup), and were reassessed during this period. This group was called the *reevaluated* group.

ID patients were included indistinctly of ID severity (mild, moderate, severe) or form of presentation (syndromic or non-syndromic). Syndromic patients were those who in addition to ID presented congenital anomalies, dysmorphic features, and/or growth disturbances.

Clinical variables were collected from medical reports, and a statistical analysis was performed looking for association with obtaining a molecular diagnosis on clinical exome sequencing.

The group of patients where a molecular diagnosis was achieved after clinical exome sequencing, were posteriorly classified in different groups according to a possible clinical diagnostic approach to evaluate the increase in the diagnostic yield due to (derived from, not possible without) clinical exome sequencing in our population.

Number and type of diagnostic procedures per patient previous to clinical exome sequencing were also collected, as well as costs derived from them in order to evaluate the economic impact of the inclusion of clinical exome sequencing in the diagnostic procedure. Other costs related to hospitalizations, imaging tests, or medical consultations of patients were not included.

We collected data on the genetic and nongenetic tests performed during patient’s diagnostic process previous to clinical exome sequencing request. All patients had CMA and fragile X molecular analyses performed, as it was an inclusion criterion. Average costs of the diagnostic tests performed prior to clinical exome sequencing were calculated. These studies were then classified as replaceable or non-replaceable by clinical exome sequencing. CMA, Fragile X molecular analysis and methylation analysis to detect imprinting defects were considered non-replaceable studies at the time of the sequencing data analyzed. Normal CMA and fragile X molecular analysis were necessary as inclusion criteria in this study, and methylation analysis were included in this group because imprinting defects cannot be readily picked up by exome sequencing. Molecular and metabolic tests were classified as replaceable studies, because monogenic diseases are potentially detected by exome sequencing.

### Clinical exome sequencing procedure

For NGS analysis, exome enrichment was achieved through capture using the SeqCap EZ MedExome Enrichment Kit, from Roche Nimblegen, followed by sequencing on a NextSeq 500 (Illumina) with an average coverage depth over 80x. Sequence alignment was performed with genome reference version NCBI37/GRCh37 (hg19). Relevant variants detected have been referenced to RefSeq genes according to their GeneBank Accesion Number. For report elaboration, variants were categorized according to ACMG recommendations [24, 25], including variants possibly explaining the phenotype and incidental findings. Most of the cases were analyzed as singletons, followed by variant segregation analysis using parental DNA when pathogenic, likely pathogenic or candidate variants of unknown significance (VUS) were detected [24, 25].

### Statistical analysis

A descriptive analysis of the variables was performed. For qualitative variables, absolute and relative frequencies as well as confidence interval were obtained, and for quantitative variables, minimum, maximum, mean and standard deviation values were calculated. Univariant analysis was performed with Chi square test for qualitative variables and U-Mann Whitney non parametric test for quantitative variables after a normality study. P values under 0.05 were considered statistically significant.

The study protocol was approved by the Institutional Research Ethics Committee (protocol number: 2019-12-7-HCUVA) and designed following the ethical principles for medical research involving human subjects of the World Medical Association declaration of Helsinki. Written informed consent for NGS procedures according to ACMG recommendations was obtained in all cases previous to obtaining DNA samples [26].

## Results

**Fig. 1:**
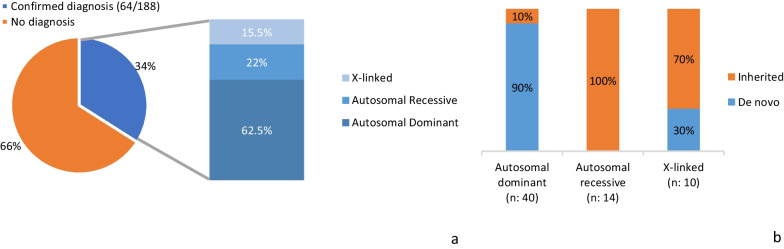
**a** Exome sequencing diagnostic yield and inheritance pattern of confirmed diagnoses. **b** Inheritance pattern of confirmed diagnoses according to type of inheritance

The diagnostic yield of clinical exome sequencing in our population was 34% (64/188) (Fig. 1a).

**Fig. 2:**
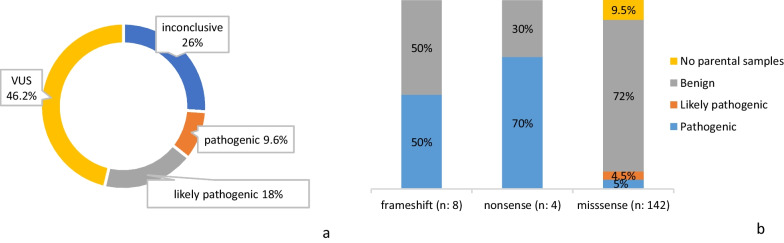
**a** Outcome of the clinical exome results. Although several variants are identified in the analysis of a clinical exome, a final result of the analysis was given after the most relevant variant identified, according to the following classification: pathogenic > likely pathogenic > VUS > inconclusive. **b** Final VUS classification according to type of variant

Pathogenic and likely pathogenic variants were detected in 27.6% of cases (52/188); and were mainly *frameshift* (36%; 19/52) and *nonsense* (34%; 18/52). 25% (13/52) were *missense* and 3.8% (2/52) *splicing variants,* and one was *nonframeshift.* A total of 157 VUS were reported in 46% of cases (87/188); which were mainly *missense* (90%; 142/157). *Frameshift* and *nonsense* VUS were detected in 5% (8/157) and 2.5% (4/157) respectively. Two *splicing* variants and one *nonframeshift* variant were also reported as VUS (Fig. 2a). After segregation analysis, 9% (14/157) of variants initially classified as VUS were finally classified as pathogenic, contributing to the final diagnostic yield in a 7.4%. Final VUS classification according to type of variant after completing molecular and pedigree investigation is shown in Fig. 2b.

Even though our clinical exome sequencing approach was a singleton approach, we performed segregation analyses on all pathogenic and likely pathogenic variants, as well as on all VUS found in suggestive candidate genes, so the final analysis of NGS results can equate to those of trio exome sequencing.

The diagnostic yield proved equal regardless of ID severity grades, or syndromic/non-syndromic ID forms, thus indicating its utility on all patients with ID independently of clinical severity. Though, there was a slightly higher percentage of diagnoses in the moderate and syndromic ID groups of patients, but without reaching statistical significance. See Table 1.

**Table 1:** Clinical exome sequencing diagnostic yield according to different patient classifications

Patient classification	Diagnostic yield (%)	*p*-value*
*ID severity*		
Mild (n = 54)	30	0.4
Moderate (n = 77)	42	
Severe (n = 41)	39	
*Syndromic / Non-syndromic ID*		
Non syndromic (n = 29)	28	0.2
Syndromic (n = 143)	39	
*Moment of evaluation*		
New patients (n = 134)	38.2	0.73
*p*-Reevaluated patients (n = 54)	34	

*Chi square test (*p*-value < 0,05)

Disease-causing mutations (65) were detected in 58 different genes, 70% of the variants were not previously described (46/65), thus highlighting the large genetic heterogeneity in ID patients. 62.5% (40/64) of the molecular diagnoses were attributable to autosomal dominant genes, and 90% (36/40) of these occurred de novo*.* See Fig. 1b.

Secondary findings according to ACMG guidelines were detected in 3.2% of patients (6/188), and carrier status of autosomal recessive genes was revealed in 15.4% of individuals (29/188). Recurrent diagnoses were detected in 12% of cases (7/58), including AHDC1 (3 patients) and EFTUD2, SHANK3, NDST1, HACE1 and MECP2 (2 patients each) which match with recurrent ID genes described by other authors. One patient with double molecular diagnosis was detected. See Additional file 1: Table 1 for description of patients with a genetic diagnosis after clinical exome sequencing.

### Clinical classification of patients with molecular diagnostic

ID patients that received a molecular diagnostic after NGS analysis we classified into several groups, according to the reason why they were (or weren’t) clinically diagnosed previous to clinical exome analysis.

37.5% (24/64) of the diagnosed patients were considered non-syndromic entities, which couldn’t be clinically addressed by clinicians. 9.4% (6/64) of the diagnoses were attributable to recently described genes, unknown until detected by NGS. 6.3% (4/64) of the diagnoses corresponded to atypical clinical presentations of known clinical entities. 18% (12/64) were classical entities which although presenting with a recognizable phenotype were not correctly guessed by clinicians. The remaining 28% (18/64) of cases were clinically addressed but achieved through clinical exome sequencing due to genetic heterogeneity or wide differential diagnosis.

In summary, more than half of the cases (53.2%) wouldn’t have been clinically addressed (and therefore not diagnosed) without the help of clinical exome sequencing.

### Time to diagnosis and time to clinical exome sequencing

Average time from first clinical examination to clinical exome sequencing analysis in all patients was 42 months with an average of 4.5 ± 3 clinical appointments. The average time to molecular diagnosis using NGS was of 4.9 ± 3.8 years. After clinical exome sequencing, the average of consultations to confirm molecular diagnosis was of 1.2 visits, necessary for obtaining simples for variant segregation analysis.

We analyzed cases in order to evaluate the impact of clinical exome sequencing in time to diagnosis of ID patients according to their classification in *new* and *reevaluated patients.* After clinical exome sequencing implementation, time to diagnosis was significantly reduced in new vs reevaluated patients with a shortening of a 60% (3.4 ± 3.05 years in the new patient group vs 8.6 ± 3.2 years in the reevaluated group; *p*-value < 0.001). Similar reduction was observed with respect to number of consultations to diagnosis (3.6 consultations in new patient group versus 6.7 in reevaluated group). Diagnostic yield was not significantly different between these two groups (*new patient* group: 38% vs 34.6% in the *reevaluated* group; *p*-value 0.7), therefore excluding any possible bias originating from old, unresolved cases, which could have increased the final diagnostic yield in this study.

### Economic analysis

We performed an economic analysis to evaluate the efficiency of the implementation of clinical exome sequencing in the diagnostic workup of ID patients.

Above 80% of patients had multiple metabolic tests performed in blood, urine, and cerebrospinal fluid. 41.5% had methylation tests performed in order to exclude imprinting defects causing ID such as Prader Willi / Angelman, Temple or Kagami-Ogata syndromes. 71% of patients had a direct molecular analysis performed, with an average of 2.3 molecular studies per patient. Of these, 43% were direct sequencing of a gene, 27% MLPA and 21% small, gene-targeted NGS panel.

**Fig. 3:**
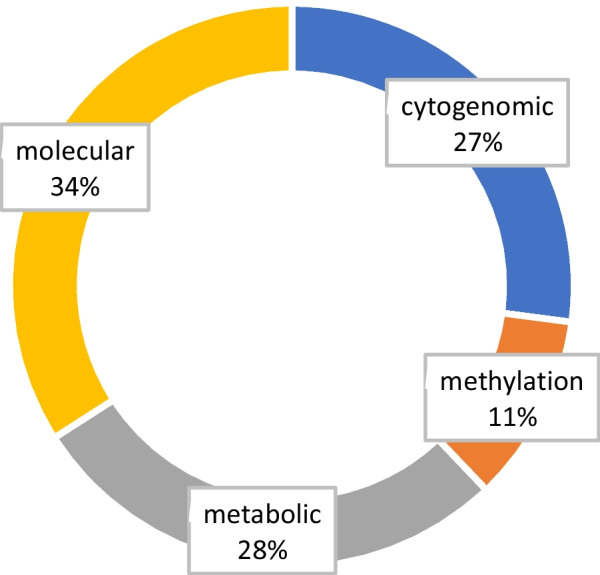
Cost distribution according to the type of tests performed previous to exome sequencing. *Cytogenomic costs* include array-based CGH and Fragile X molecular analysis and karyotype or FISH when performed. *Molecular costs* include direct gene sequencing, gene MLPA, and gene-targeted panels

Average cost related to diagnostic workup performed previous to clinical exome sequencing was 2.702 ± 1.385 euros per patient. The distribution of cost according to the type of test is shown in Fig. 3. The “cytogenomic” category includes the costs of CMA, and other chromosomal studies (karyotype and FISH) as well as Fragile X molecular analysis in order to minimize categories.

Average costs of clinical exome sequencing was 1.100 ± 300 euros per patient. The cost of Sanger analyses for segregation analyses of detected variants, which were performed in 74.5% of cases, secondary to VUS segregation or inheritance pattern verification in the case of pathogenic or likely pathogenic variants, was included.

After clinical exome sequencing analysis, other genetic tests were considered necessary in 5.3% of the patients. These studies were mostly to determine skewed patterns of X-inactivation in cases of detection of a causal variant in X-linked genes. Another common genetic test was methylation analysis that was performed in negative clinical exome patients, where phenotype suggested this possibility in an effort to achieve a diagnosis. These costs added up to an average of 266 euros/patient.

The average overall direct costs of the diagnostic procedure in ID patients in our population and setting (considering pre-exome costs and clinical exome costs) was about 4.000 euros per patient.

**Fig. 4:**
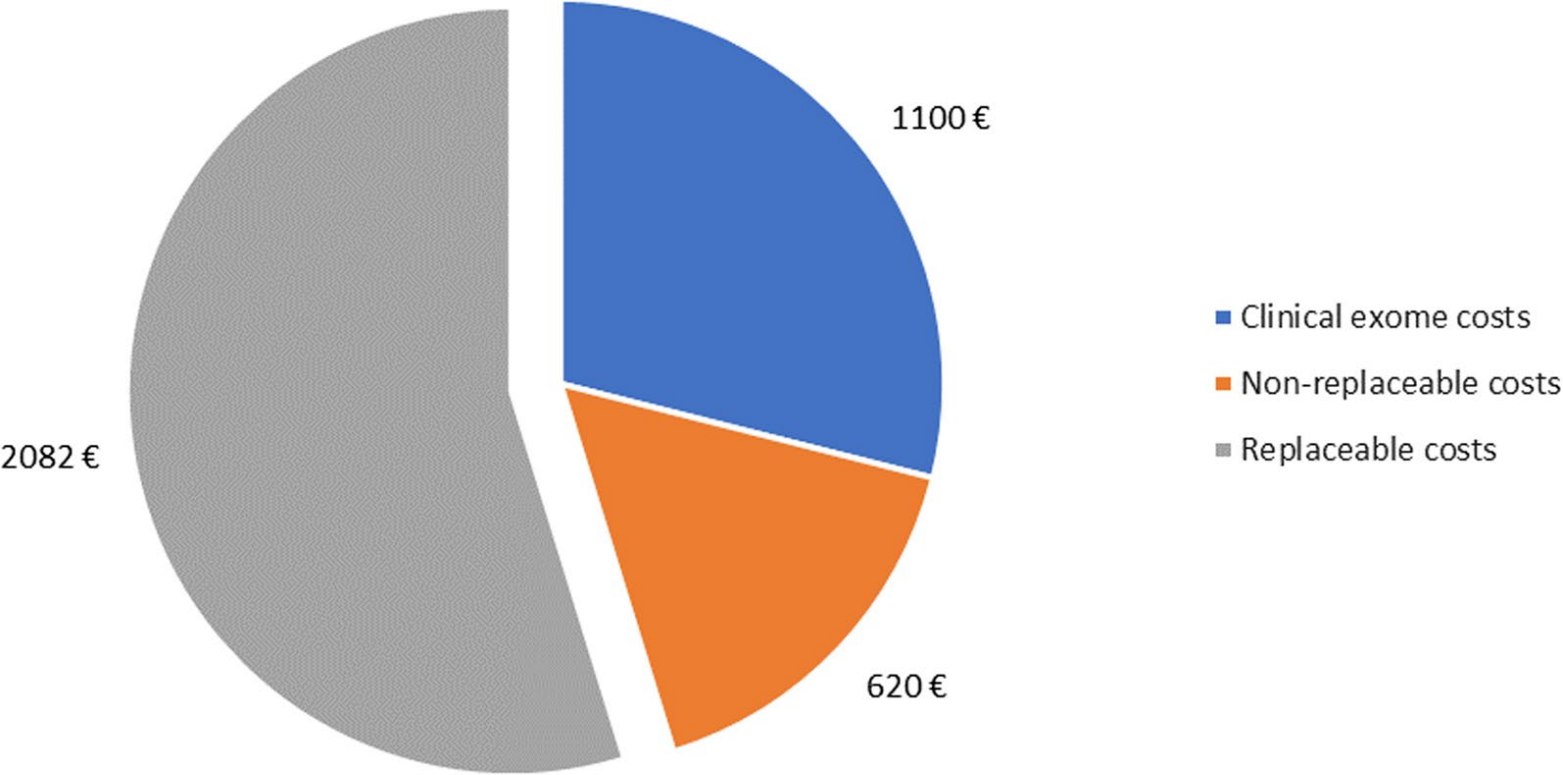
Cost distribution of genetic analysis in patients with intellectual disability. *Replaceable costs* referred to molecular and metabolic studies / *Non-replaceable* costs referred to cytogenomic, fragile-X molecular analyses and methylation analysis

Using the above-mentioned clasification of NGS-replaceable and nonreplaceable tests, 62% of costs previous to clinical exome sequencing came from metabolic and molecular tests, which could be replaced by it. Thus, we calculated that using clinical exome sequencing after CMA and fragile X molecular analysis in ID patients would have produced average savings worth 2.082 ± 1.191 euros per patient (see Fig. 4).

With respect to genetic tests after clinical exome sequencing, we observed a reduction of 80.7% of average costs in ID patients respect to the costs of test performed previous to clinical exome sequencing.

**Fig. 5:**
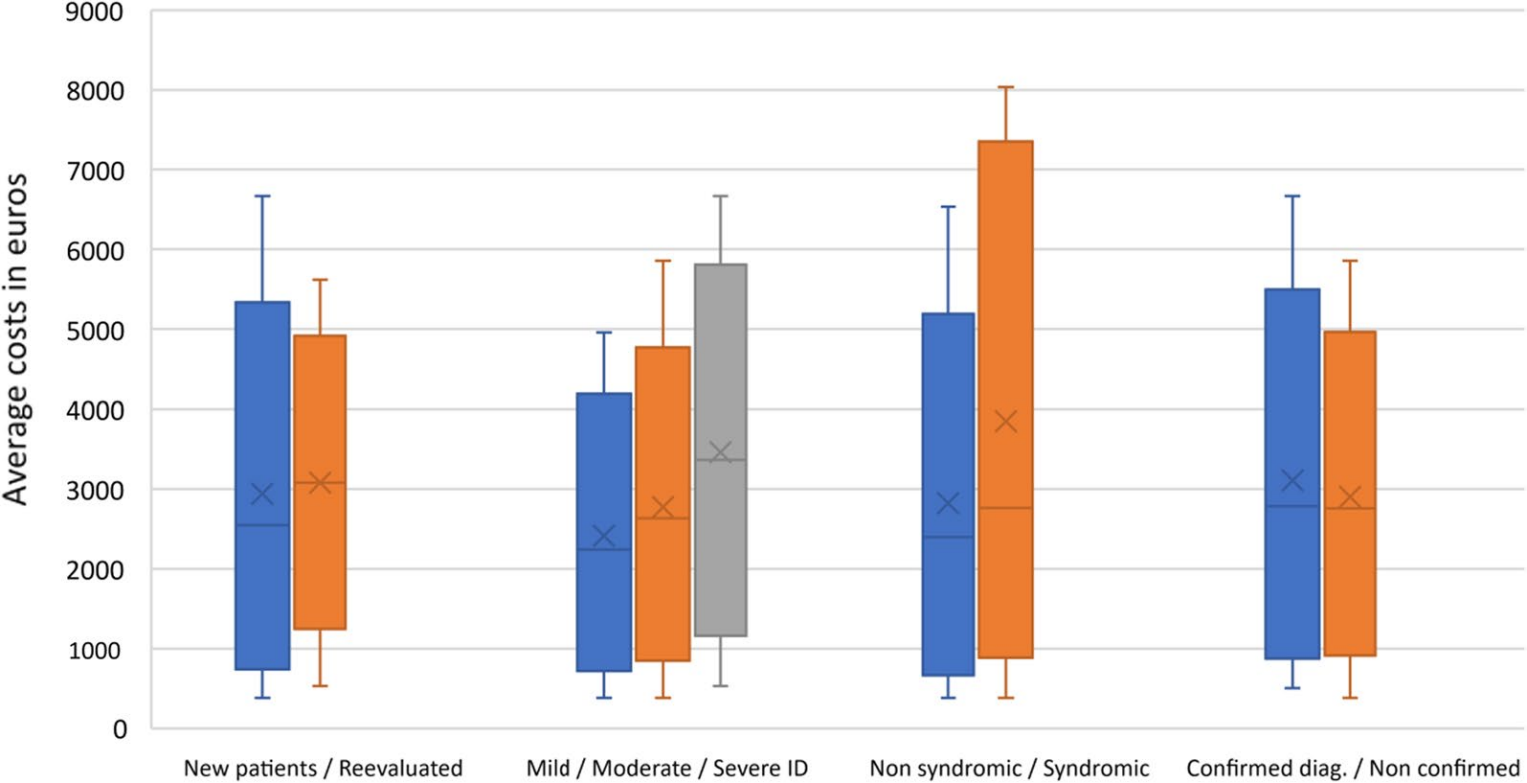
Average costs of the test performed previous to clínical exome sequencing in ID patients according to the different classification of patients in this study. No statystical differences were detected

We also analyzed the average costs of the test performed previous to clinical exome sequencing in ID patients according to different classification criteria of patients in this study. No statistical differences were detected according to average costs in any of these classifications (see Fig. 5). Note that no differences were detected according to classification of patients with a final molecular diagnosis of those without a diagnosis, which supports the *end of trajectory effect* described after exome sequencing application even if you reach a diagnosis or not.

## Discussion

Diagnostic yield of clinical exome sequencing was significantly high (34%) and similar to reported yields in other studies, supporting its utility in molecular diagnosis of ID patients in clinical settings. Different articles show similar diagnostic yields in ID patients after using exome or genome sequencing [6, 10, 13, 20, 27–30], supporting clinical exome sequencing as an effective approach for clinical practice for the moment. After these results, we suggest focusing on the analysis of the clinical exome due to the easier interpretation of detected variants, and the difficulties that we envision in a clinical setting to carry out functional analyses of potentially pathogenic variants detected in ES or GS approaches outside of genes with a well-established relationship with disease. That being said, in a short time, with the increasingly fast gain of understanding of the exome and the enrichment of databases with new described pathogenic variants will probably make ES an adequate approach in clinical practice.

Even though our clinical exome sequencing approach was directed to singleton index cases, we performed segregation analyses of all pathogenic, likely pathogenic variants as well as all VUS in suggestive candidate genes, so the final analysis of NGS results can equate to those of trio exome sequencing. The contribution of 7.4% to the diagnostic yield after VUS analysis and final classification reinforces the need of VUS examination, selection and analysis when indicated. In our study around 74% of patients needed segregation analysis in parents, secondary to VUS interpretation or to check inheritance pattern of pathogenic or likely pathogenic variants. A trio-based exome analysis will obviate this need, but its costs are for the moment higher, so these numbers must be analyzed in order to establish the most efficient approach. Another arguably benefit of using a trio-based approach would have been a more straightforward discovery of de novo variants in dominant genes.

Respect to clinical variables in patients which could suggest a higher probability to have a molecular result in clinical exome sequencing, no association was found between any of the variables analyzed and a higher diagnostic yield. This indicates clinical exome sequencing should be performed in all ID patients indistinctly of clinical presentation after CMA and fragile X molecular analysis.

Our results reveal that de novo autosomal dominant mutations are the most frequent cause in ID patients, as described in the literature [6, 18, 20, 31, 32], which explains why incidence of ID remains stable despite improvement of diagnosis and genetic counselling of ID families, as suggested by other authors [3]. 65 variants were detected in 58 different genes, 70% of which were new pathogenic variants not previously described in the literature, supporting genetic heterogeneity in ID patients. Recurrent genes in our series overlap with those described by other authors [11, 14, 17, 18, 29, 31], as well as incidental findings and carrier status detection [6, 20, 28, 31].

### Diagnostic tests previous to clinical exome sequencing

The individuals included in this study are a selected group of patients in which a possible exogenous, non-genetic cause of ID was excluded after an exhaustive and complete clinical evaluation. The absence of chromosomal imbalances and fragile X syndrome were also inclusion criteria. These are common inclusion criteria in many studies which evaluate WES and WGS in ID patients [13, 17, 33–35], which is obvious because these analyses rule out common genetic causes of ID, which at least in the beginning would not be detected by NGS. In the last years, different articles report on the feasibility of CNV detection using NGS data, which will allow a unified test for sequence and copy number variant detection in a near future [20, 36] which will obviously have an impact in the diagnostic costs of ID patients.

Most of the patients had other tests performed looking for possible genetic causes to explain their ID, previous to clinical exome sequencing request, such as metabolic, methylation and other gene-centric molecular tests. Respect to metabolic testing, they have been suggested as first line testing in patients with ID, due to the fact that these illnesses are potentially treatable and can benefit from an early diagnosis [4, 13, 23, 37]. Metabolic disorders represent up to 1–5% of patients with ID [38]. In the last years, some authors suggest the possibility to perform a molecular approach to these diseases if there is a good access to NGS in the clinic, in a way to obviate the multiple and expensive biochemical metabolic tests [2, 22, 39]. Other authors also evaluated the impact of NGS in the diagnosis of metabolic diseases with atypical forms of clinical and metabolic presentations, as well as for the detection of new candidate genes [7, 40]. In our study we detected a case of Smith-Lemli-Opitz syndrome which had an atypical clinical presentation, with normal cholesterol serum levels and slightly increased sterols, as well as a patient with congenital disorder of glycosylation type 1A and normal sialotransferrins (unpublished personal communication). Therefore after review of the literature and our own findings, we suggest that once the most common and treatable metabolic diseases are ruled out in the neonatal screening test, diagnosis of metabolic diseases could be approached with NGS.

Respect to methylation tests to rule out epigenetic-imprinting diseases, which are not caused by permanent change in the DNA sequence, those will still be necessary because they cannot be detected by clinical exome sequencing [8]. Epigenetic causes have been described as cause for ID [7], classical entities as Prader-Willi or Angelman syndromes, but also more recent entities such as Temple or Kagami-Ogata syndromes. Some authors highlight how after NGS, diagnosis as Silver-Russell syndrome or DUP14 had not been detected until direct analysis was indicated [8].

In our population 80% of patients had a gene-targeted molecular analysis performed previous to clinical exome sequencing with an average of 2.3 molecular tests per patient. This data is similar to that published in the literature which describe 2–3 molecular tests per patient prior to NGS [7, 8].

### Time to diagnosis

These results prove the impact of using clinical exome sequencing in ID patients as to a shorter time to diagnosis. The shorter time to diagnosis as well as the fewer clinical consultations in the group of *new patients* which had access to NGS from the beginning shows the impact of NGS in the *diagnostic odyssey* of ID patients with multiple clinical appointments, multiple testings without reaching a final diagnosis described in the literature [7]. In the same way, in this study a decline in testing and consultations after clinical exome sequencing were also observed wether or not a diagnosis had been reached, supporting the *final trajectory effect* [23].

The 17 patients which achieved a diagnosis in the *reevaluated group* could’ve have been diagnosed before if NGS had been available, with the impact this would’ve had in their sanitary, social, educational and familiar spheres [38].

### Costs of-analysis

ID healthcare costs are high, equating costs of other pathologies such as cerebral stroke, ischemic heart disease or cancer [38]. Some authors estimate lifespan costs of around 900.000 euros for ID patients, including costs referred to medical consultations, hospital admissions, sanitary transportation as well as diagnostic studies such as imaging or analytic analyses [22, 23]. Other authors focus on the economic impact of the diagnostic procedure of ID patients estimating about 14–15.000 euros per patient. These authors estimate that around 40% of these costs belong to genetic studies (4.000–5.000 euros on average) [22], which match with the costs calculated in this study.

In this study, we have performed an economic analysis focusing in the costs derived from the genetic studies performed during the diagnostic process of ID patients in order to evaluate the impact and efficiency of introducing clinical exome sequencing earlier in the diagnostic workup.

The efficiency of using clinical exome sequencig as first option after normal CMA and Fragile X molecular analysis in ID patients has been shown and proved after the economic analysis in this study, estimating saving of 2082 euros per patient, which represents a 62% of savings respect to previous genetic studies performed in ID patients. Other authors estimate similar savings concerning genetic studies, of around 774–2300 euros per patient after NGS in ID patients [16, 22, 23, 30]. These authors also mention the effect of the *end of trajectory effect* of exome sequencing in ID patients independently of the result obtained [23]. The comparison of pre and post exome sequencing costs show a reduction of 80% of the latter, as well as a reduction of 50% in clinical consultation and analytic studies. This *end of trajectory effect* is also observed in our study with a pre-exome sequencing expenses of 2.702 euros per patient, and an average post-exome sequencing expenses of 266 euros per patient, which corresponds to an 80.7% reduction. These results support the fact suggested by other authors that using exome sequencing sooner in the diagnostic procedure does not only reduce the costs of the diagnostic procedure, but also the cost of other genetic tests performed afterwards independently of the obtained diagnosis.

After analyzing the results obtained in this study, the increased diagnostic yield as well as the fact that many of the diagnoses weren’t clinically detectable, the reduction of time to diagnosis and the economic savings with respect to classical diagnostic studies, strengthen the value of early implementation of clinical exome sequencing in the diagnostic process of ID patients in clinical practice. We propose a new, more effective and efficient, diagnostic algorithm for ID patients in our population (see Additional file 2: Fig. 1).

The main objective of this study was to investigate the diagnostic yield of clinical exome sequencing in a group of ID patients in which other nongenetic causes of ID, as well as common chromosomal, imprinting and clinically recognizable genetic entities had previously been ruled out. In other words, good candidate patients for a monogenic cause of ID. Some studies define a correct etiologic diagnosis after first medical genetic consultation in 17–33% of cases due to. non-genetic, exogenous causes as well as clinically assesed genetic causes [41]. In our study we haven’t collected the non-genetic or clinically adresssed diagnoses in our Section in first consultation, and therefore this is not a study of the causes of ID in our population, but in a specific group of patients as described earlier.

Another possible confounding factor is that ID severity of patients in our cohort does not follow the usual distribution seen in the general population, with a higher representation of moderate to severe and syndromic ID patients. This is due to the fact that patients referred to our consultation have been previously adressed by neurologists which usually refer to us patients with a more severely affected IQ or syndromic forms in order to search for a possible genetic cause. Anyway, statistical analysis showed no differences in diagnostic yield of clinical exome sequencing in the different ID severity groups or in isolated vs. syndromic ID, supporting the usefulness of NGS approach in all cases.

### Future investigations

Achieving a molecular diagnosis in ID patients has an impact not only in the patient care and in its family derived from adequate genetic counselling, but also in healthcare, educational and at social level. Therefore, improvement in genetic diagnostic techniques as well as their incorporation in the healthcare system should be a priority for policy makers. Professionals who are concerned and work in the diagnosis of etiological causes of ID patients must continue their investigations towards elucidating the genetic causes of ID.

In this sense, the group of patients which have not received a molecular diagnosis after initial NGS analyses, are a perfect group to continue with genomic investigations. Literature describes an improvement of diagnostic yield after exome reanalysis of around 15–41% thanks to the integration of new knowledge sources and analysis methods over time [19, 42], therefore this could be an initial procedure in these patients, especially in those in which clinical exome analysis was performed in the first years of this study. These patients and their families might also benefit from being included in a research setting using ES o GS in order to detect new ID genes.

Molecular diagnostic technologies are developing in an exponential way, which is changing the paradigm of genetic diseases, as well as diagnostic procedures and treatment of genetic disease in medicine. As many authors have previously mentioned [38, 43] this new paradigm of healthcare requires a multidisciplinary approach brinding together different areas of expertise brought by molecular and clinical geneticists, to deliver the best of NGS technology for the benefit of ID patients. Functional studies of VUS detected in NGS, exhaustive and homogeneous clinical phenotyping through HPO terms, and national and international collaboration are essential items to advance in the knowledge of new molecular causes of ID.

## Conclusions

The diagnostic yield of clinical exome sequencing in patients with intellectual disability in our population is of 34%, similar to diagnostic yields described in the literature for clinical exome sequencing, WES and WGS, which favors its priorization as diagnostic tool.

Our study proves genetic heterogeneity in ID, with detection of causal variants in 59 different genes associated with ID, of which 63.5% are new variants, not previously described in the literature.

The majority of variants detected are loss of function variants in autosomal dominant genes (62.5%), mostly being de novo in affected patients (90%).

VOUS segregation analysis increased the diagnostic yield in 7.4%, which shows the need of a correct interpretation and classification in case of singleton approach.

No statistically significant correlation was detected between clinical characteristics of patients and diagnostic yield, and 53.2% of diagnosis couldn’t be clinically directed, which prompts its use as a first tier diagnostic tool in patients with ID.

Efficiency of clinical exome sequencing in ID patients has been proven, with savings about 50% respect to regular diagnostic approach.

These results have allowed the performance of an efficient and consistent clinical diagnostic guide for ID patients in our population, which can be extrapolated to other populations, and systematically reviewed in the future.

## Supplementary Information

**Additional file 1** Table 1: Clinical and molecular data of diagnosed patients after clinical exome sequencing

**Additional file 2** Figure 1: Proposed diagnostic algorithm for ID patients

## Data Availability

The authors confirm all data is fully available, without restrictions. Data in this document has not been shared previously.
