# Supplementary material for: Validation of clinical exome sequencing in the diagnostic procedure of patients with intellectual disability in clinical practice
Source: Orphanet J Rare Dis. 2023 Jul 21;18:201. doi: 10.1186/s13023-023-02809-z (PMC10362575; doi:10.1186/s13023-023-02809-z)
Supplement: Supplementary file 1 — Additional file 1 Table 1: Clinical and molecular data of diagnosed patients after clinical exome sequencing [file 13023_2023_2809_MOESM1_ESM.pdf]

Gene †	Locus	Pathogenic variant ‡	Associated syndrome	OMIM§
**Autosomal Dominant: inherited**				
RAD21 / 606462	8q24.11	NM_006265: c.589C>T, p.Gln197Ter mat	Cornelia de Lange syndrome 4 with or without midline brain defects	614701
SUFU / 607035	10q24.32	NM_016169: c. 157C>T, p.Gln53Ter mat	Basal cell nevus syndrome	109400
NF1 / 613113	17q11.2	NM_000267: c.3437T>G, p.Val1146Gly mat	Neurofibromatosis-Noonan syndrome	601321
SCN2A / 182390	2q24.3	NM_001040143: c.3631G>A, p.Glu1211Lys mat (mosaic)	Developmental and epileptic encephalopathy 11	613721
**Autosomal Dominant: *de novo***				
AHDC1 / 615790	1p36.1	NM_001029882: c.2062C>T, p.Arg688Ter	Xia-Gibbs syndrome	615829
AHDC1 / 615790	1p36.1	NM_001029882: c.2565del, p.Phe855LeuFsTer	Xia-Gibbs syndrome	615829
AHDC1 / 615790	1p36.1	NM_001029882: c.2260T>C, p.Gln754Ter	Xia-Gibbs syndrome	615829
EFTUD2 / 603892	17q21.31	NM_001258354: c.2531+1G>A	Mandibulofacial dysostosis, Guion-Almeida type	610536
EFTUD2 / 603892	17q21.31	NM_001142605: c.560C>G, p.Ser187Ter	Mandibulofacial dysostosis, Guion-Almeida type	610536
SHANK3 / 606230	22q13.33	NM_033517: c.3382_3383del, p.Leu1128fs	Phelan-McDermid syndrome	606232
SHANK3 / 606230	22q13.33	NM_033517: c.2246dup; p.Leu749fs	Phelan-McDermid syndrome	606232
KANSL1 / 612452	17q21.31	NM_001193466: c.1816C>T, p.Arg606Ter	Koolen-De Vries syndrome	610443
KCNB1 / 600397	20q13.13	NM_004975: c.1230del, p.Phe410fs	Developmental and epileptic encephalopathy 26	616056
MBD5 / 611472	2q23.1	NM_018328: c.2190del, p.Leu730fs	Mental retardation, autosomal dominant 1	156200
GRIN2B / 138252	12p13.1	NM_000834: c.2459G>C, p.Gly820Ala	Mental retardation, autosomal dominant 6, with or without seizures	613970
EP300 / 602700	22q13.2	NM_001429: c.5798_5799del, p.Gln1933fs	Rubinstein-Taybi syndrome 2	613684
ZBTB18 / 608433	1q44	NM_205768: c.943_944del, p.Arg315fs	Mental retardation, autosomal dominant 22	612337
ARID1B / 614556	6q25.3	NM_020732: c.5356A>T, p.Lys1786Ter	Coffin-Siris síndrome 1	135900
KAT6B / 605880	10q22.2	NM_001256468: c.2474_2475ins, p.Ala825fs	Ohdo syndrome, SBBYS variant	603736
SMC1A / 300040	2q24.3	NM_006306: c.1117A>T, p.Lys373Ter	Developmental and epileptic encephalopathy 85 with or without midline brain defects	301044
ACTB / 102630	7p22.1	NM_001101: c.547C>T, p.Arg183Trp	Baraitser Winter síndrome 1	243310
SHANK2 / 603290	11q13.3	NM_133266: c.902dup, p.Pro302fs	Susceptibility to Autism, 17	613436
PURA / 600473	5q31.3	NM_005859: c.678del, p.Val226fs	Mental retardation, ausotomal dominant 31	616158
STXBP1 / 602926	9q34.11	NM_001032221: c.504del, p.Ile168fs	Developmental and epileptic encephalopathy 4	612164
GRIN1 / 138249	Pq34.3	NM_000832: c.2413C>T, p.Pro805Ser	Neurodevelopmental disorder with/without hyperkinetic movements and seizures, autosomal dominant	614254
SMAD4 / 600993	18q21.2	NM_005359: c.1498A>G, p.Ile500Val	Myhre syndrome	139210
ZEB2 / 605802	2q22.3	NM_001171653: c.3099T>A, p.Cys1033Ter	Mowat-Wilson syndrome	235730
RAF1 / 164760	3p25.2	NM_002880: c.782C>T, p.Pro261Leu	Noonan síndrome 5	611553
SYNGAP1 / 603384	6p21.32	NM_006772: c.2895del, p.His965fs	Mental retardation, autosomal dominant 5	612621
HNRNPK / 600712	9q21.32	NM_002140: c.1347T>G, p.Tyr449Ter	Au-Kline syndrome	616580
KMT2A / 159555	11q23.3	NM_001197104: c.7255G>T, p.Glu2419Ter	Wiedemann-Steiner syndrome	605130
CTNNB1 / 116806	3p22.1	NM_001098209: c.1603C>T, p.Arg535Ter	Neurodevelopmental disorder with spastic diplegia and visual defects	615075
NSD1 / 606681	5q35.3	NM_022455: c.3964C>T, p.Arg1322Ter	Sotos síndrome 1	117550
TCF4 / 602272	18q21.2	NM_001243234: c.1017dup, p.Gly340fs	Pitt-Hopkins syndrome	610954
CDK13 / 603309	7p14.1	NM_003718: c.2525A>G, p.Asn842Ser	Congenital heart defects, dysmorphic facial features, and intellectual developmental disorder	617360
CHD2 / 602119	15q26.1	NM_001271: c.5225G>A; p.Arg1742Gln	Developmental and epileptic encephalopathy 94	615369
AFF4 / 604417	5q31.1	NM_014423: c.772C>T; p.Arg258Trp	CHOPS syndrome	616368
KMT2B / 606834	10q13.12	NM_014727: c.4903C>T, p.Arg1635Ter	Dystonia 28, childhood-onset	617284
KCNQ2 / 602235	20q13.33	NM_004518: c.913_915del; p.Phe305del	Developmental and epileptic encephalopathy 7	613720
SOX10 / 602229	22q13.1	NM_006941: c.743_744del, p.Glu248fs	Waardenburg syndrome, type 2E	611584

Gene †	Locus	Pathogenic variant ‡	Associated syndrome	OMIM§
**Autosomal Recessive**				
NDST1 / 600853	5q33.1	NM_001301063: c.1831G>A; p.Gly611Ser homozygosity	Mental retardation, autosomal recessive 46	616116
NDST1 / 600853	5q33.1	NM_001301063: c.1831G>A; p.Gly611Ser homozygosity	Mental retardation, autosomal recessive 46	616116
HACE1 / 610876	6q16.3	NM_020771: c.697_703del; p.Leu233fs homozygosity	Spastic paraplegia and psychomotor retardation with or without seizures	616756
HACE1 / 610876	6q16.3	NM_020771: c.587C>G, p.Ser196Ter homozygosity	Spastic paraplegia and psychomotor retardation with or without seizures	616756
LRP5 / 603506	11q13.2	NM_002335: c.1282C>T, p.Arg428Ter homozygosity	Osteoporosis-pseudoglioma syndrome	259770
DHCR7 / 602858	11q13.4	NM_001163817: c.452G>A, p.Trp151Ter / c.1A>G, p.Met1Val	Smith-Lemli-Opitz syndrome	270400
OTUD6B / 612021	8q21.3	NM_016023: c.433C>T, p.Arg145Ter homozygosity	Intellectual developmental disorder with dysmorphic facies, seizures, and distal limb anomalies	617452
LAMA1 / 150320	18p11.31	NM_005559: c.5369del; p.Arg1790fs / c.2935del; p.Arg979fs	Poretti-Boltshauser syndrome	615960
AHI1 / 608894	6q23.3	NM_017651: c.2488C>T, p.Arg830Trp / c.1-22T>C	Joubert syndrome 3	608629
CSPP1 / 611654	8q13.1	NM_001291339: c.1979C>G, p.Thr660Ser homozygosity	Joubert syndrome 21	615636
LARS2 / 604544	3p21.31	NM_015340: c.1565C>A, p.Tht522Asn / c.632A>T, p.Asp211Val	Perrault syndrome 4	615300
DENND5A / 617278	11p15.4	NM_015213: c.1011G>A, p.Trp337Ter homozygosity	Developmental and epileptic encephalopathy 49	617281
ERCC3 / 133510	2q14.2	NM_000122: c.1631G>A; p.Cys544Tyr homozygosity	Trichothiodystrophy 2, photosensitive	616390
MASP1 / 600521	3q27.3	NM_139125: c.199G>A; p.Gly665Ser homozygosity	3MC síndrome 1	257920
**X-linked**				
MECP2 / 300005	Xq28	NM_001110792: c.459C>G, p.Tyr153Ter	Rett syndrome	312750
MECP2 / 300005	Xq28	NM_001110792: c.313C>T; p.Pro105Ser	Rett syndrome	312750
PIGA / 311770	Xp22.2	NM_002641: c. 348A>G, p.Ile116Met	Multiple congenital anomalies-hypotonia-seizures syndrome	300868
SLC9A6 / 300231	Xq26.3	NM_001042537: c.1176_1177; p.Gln394ArgfsTer6	Mental retardation, X-linked, syndromic, Christianson type	300243
IQSEC2 / 300522	Xp11.22	NM_001111125: c.3470A>C; p.Asn1157Thr	Mental retardation, X-linked 1	309530
DDX3X / 300160	Xp11.4	NM_001193417: c.1939_1940del, p.*647fs	Intellectual developmental disorder, X-linked, syndromic, Snijders Blok type	300958
RPS6KA3 / 300075	Xp22.12	NM_004586: c.53del; p.Pro18ArgfsTer38	Coffin-Lowry syndrome	303600
AFF2 / 300806	Xq28	NM_001170628: c.932G>A; p.Arg311His	Intellectual developmental disorder, X-linked 109	309548
MED12 / 300188	Xq13.1	NM_005120: c.6440A>G; p.Gln2147Arg	Opitz-Kaveggia syndrome	305450
OPHN1 / 300127	Xq12	NM_002547: c.313-1G>T	Mental retardation, X-linked, with cerebellar hypoplasia and distinctive facial appearance	300486
KLHL15 / 300980	Xq22.11	NM_030624: c.417_418del, p.Leu139fs	Mental retardation, X-linked 103	300982

† HGNC-approved gene symbols and OMIM database reference number for the gene
‡ Reported variants: GeneBank Accession Number (RefSeq) / Sequence variant nomeclature according to HGVS guidelines (DNA and protein designations)
§ OMIM database reference number for the disorder

Supplementary Table 1: Details on the clinical and molecular diagnosis of patients included in the study.
