# Supplementary figures and images for: Validation of clinical exome sequencing in the diagnostic procedure of patients with intellectual disability in clinical practice
Source: Orphanet J Rare Dis. 2023 Jul 21;18:201. doi: 10.1186/s13023-023-02809-z (PMC10362575; doi:10.1186/s13023-023-02809-z)

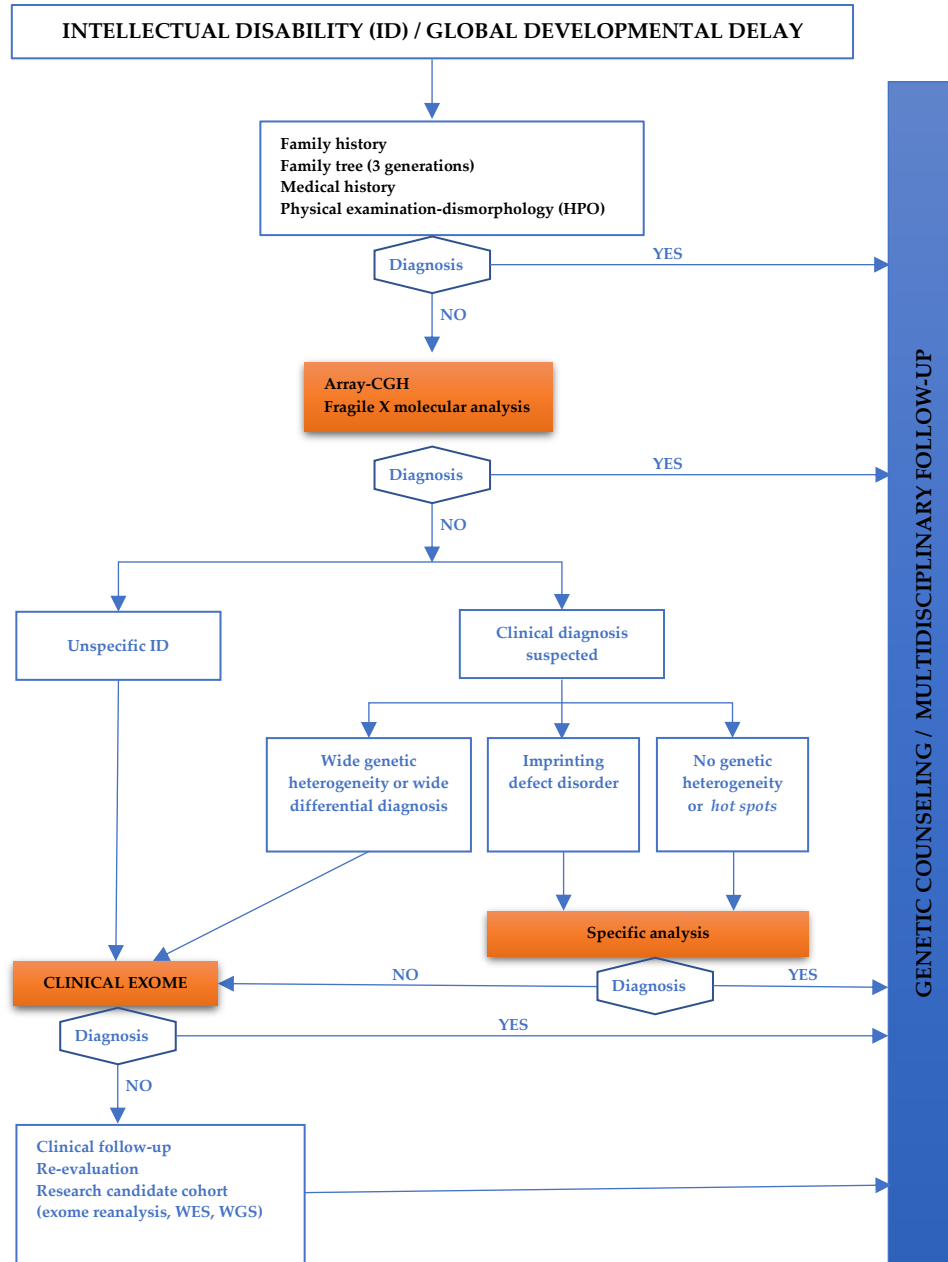

Supplement: Supplementary file 2 — Additional file 2 Figure 1: Proposed diagnostic algorithm for ID patients [file 13023_2023_2809_MOESM2_ESM.pdf]
